# Multimodal Approach Combining Thulium Laser Vaporization, Bipolar Transurethral Resection of the Prostate, and Bipolar Plasma Vaporization versus Bipolar Transurethral Resection of the Prostate: A Matched-Pair Analysis

**DOI:** 10.3390/jcm13164863

**Published:** 2024-08-17

**Authors:** Roxana Andra Coman, Radu Tudor Coman, Răzvan-Ionuț Popescu, Daniel Corneliu Leucuta, Răzvan Couți, Ioan Coman, Nadim Al Hajjar

**Affiliations:** 1 Department of Urology, “Iuliu Hatieganu” University of Medicine and Pharmacy, 400012 Cluj-Napoca, Romania; dr.roxanacoman@yahoo.com (R.A.C.);; 2 Department of Urology, Endoplus Clinic, 400165 Cluj-Napoca, Romania; 3 Department of Epidemiology, “Iuliu Hatieganu” University of Medicine and Pharmacy, 400347 Cluj-Napoca, Romania; 4 Department of Urology, “Carol Davila” University of Medicine and Pharmacy, 050474 Bucharest, Romania; 5 Department of Urology, “Prof. Dr. Th. Burghele” Clinical Hospital, 061344 Bucharest, Romania; 6 Department of Medical Informatics and Biostatistics, “Iuliu Hatieganu” University of Medicine and Pharmacy, 400349 Cluj-Napoca, Romania; 7 Bistrita County Emergency Hospital, 420016 Bistrita, Romania; 8 Department of Surgery, “Iuliu Hatieganu” University of Medicine and Pharmacy, 400012 Cluj-Napoca, Romania; 9 Department of Surgery, Regional Institute of Gastroenterology and Hepatology “Octavian Fodor”, 400394 Cluj-Napoca, Romania

**Keywords:** BPH, Thulium laser, combined technique, bipolar TURP

## Abstract

**Background/Objectives:** The aim of our study is to compare the perioperative and functional outcomes of a multimodal approach combining thulium laser vaporization, bipolar TURP, and bipolar plasma vaporization (TLP) with bipolar TURP in a matched-pair analysis. **Methods:** A nonrandomized, observational, retrospective, and matched-pair analysis was performed on two homogeneous groups of 60 patients who underwent TLP versus bipolar TURP at our center between March 2018 and December 2021. The American Society of Anesthesiologists (ASA) score and prostate volume (PV) were the main parameters used to match patients between the two groups. Follow-up was evaluated at 3, 6, 12, and 24 months after surgery. **Results:** There was a shorter operative time in favor of TLP (42 versus 45 min, *p* = 0.402). Median hemoglobin drop (−0.3 versus −0.6, *p* < 0.001) and median sodium drop (−0.3 versus −0.7, *p* < 0.001) after surgery were statistically significantly lower in TLP compared to bipolar TURP. The International Prostate Symptom Score (IPSS) and Quality of Life (QoL) scores were significantly lower, and the maximum urinary flow rate was higher in the TLP group. The median PSA decrease 2 years after surgery was 73.92% in the TLP group versus 76.17% in the bipolar TURP group (*p* = 0.578). The complication rate was lower in the TLP group (20% versus 21.67%, *p* = 1). **Conclusions:** The results show that both procedures are equally effective and safe in the treatment of symptomatic BPH with some advantages regarding the TLP technique.

## 1. Introduction

For many decades, the transurethral resection of the prostate (TURP) has been the gold-standard surgical treatment for lower urinary tract symptoms (LUTS) related to benign prostatic hyperplasia (BPH) [1]. Bipolar TURP offers the same efficiency in improving urinary symptoms as traditional monopolar TURP. However, it also has important advantages, such as working in a saline environment and providing better hemostasis. This allows surgeons to safely operate on larger prostates with a low risk of TUR syndrome and reduced transfusion rates [2]. After introducing Thulium laser therapy, various techniques have been created that combine vaporization and resection to remove the prostatic adenoma without requiring direct access to the prostatic capsule [3]. Enucleation techniques use blunt mechanical dissection or vaporization to remove the prostatic lobes from the prostatic capsule, creating an anatomical enucleation plane [4].

The Thulium laser is effective in the processes of coagulation, vaporization, and hemostasis in tissues. Water absorbs its high energy, resulting in thermal damage primarily in the surface structure, which restricts the tissue’s energy penetration depth [5]. Compared to TURP, these techniques have been shown to have similar improvements in functional outcomes, with lower transfusion rates and shorter hospitalization and catheterization times. However, TURP had a significantly lower operation time [6]. These findings remained consistent even for prostates with a high volume and were more effective when compared to TURP [7]. The incidence of temporary urine incontinence was greater in Thulium laser vapo-resection (ThuVARP) compared to TURP (20.9% versus 4.7%) [8]. According to the available evidence, the plasmakinetic resection of the prostate (PKRP) is more effective than TURP in terms of transfusion rate, catheterization, and operative time [9]. In a long-term follow-up, TURP had better functional outcomes compared to the plasmakinetic vaporization of the prostate (PKVP). However, PKVP had better results in decreasing hemoglobin levels [10].

Because the surgical treatment of BPH requires long-term follow-up, recent advances in telemedicine have significantly improved post-operative care and monitoring for BPH treatments, offering innovative tools for patient management outside the clinical setting, such as a low-cost home-based uroflowmetry device that facilitates the remote monitoring of urinary flow rates for efficient follow-up protocols [11].

Huang et al. investigated the association between Thulium laser and bipolar TURP compared to bipolar TURP. The study included a moderate number of patients and reported comparable perioperative outcomes. However, the combined technique was found to have a higher resection efficiency, particularly in cases of prostate volumes ≥40 g. It is important to note that this study only reported a short follow-up period of 2 months [12]. Xie et al. found that the combination of a Thulium laser with PKRP provided better outcomes for patients with large prostate volumes (>80 mL) compared to PKRP alone. The results showed an improved operative time, reduced blood loss, increased efficiency, and faster postoperative recovery at 3 months follow-up [13].

We intended to combine the benefits of these techniques while minimizing their drawbacks. To achieve this, we developed a multimodal approach that combines Thulium laser vaporization, bipolar TURP, and plasma vaporization. We named our technique TLP. The aim of our study is to compare the perioperative and functional outcomes of our technique (TLP) to bipolar TURP in a matched-pair analysis.

## 2. Materials and Methods

This study was an observational, retrospective, and matched-pair analysis. It was a controlled study, but not a randomized one. The study analyzed data that were prospectively obtained from a series of patients who underwent TLP or TURP for symptomatic LUTS between March 2018 and December 2021. The study was reviewed by the Ethics Committee of the “Iuliu Hațieganu” University of Medicine and Pharmacy under reference number 236/20 September 2023. The study was conducted in accordance with the ethical principles of the Declaration of Helsinki for medical research involving human subjects. All patients included their data in the database and consented to its use for scientific research purposes.

The study was conducted in a single center. Comparability was ensured by a careful review of clinical databases for prostate volume (PV) and the American Society of Anesthesiologists (ASA) score.

### 2.1. Surgical Indication

The indication for surgery was a symptomatic BPH refractory to medical therapy. Regardless of PV, patients were recommended surgery if they had a maximum urinary flow rate (Qmax) of less than 15 mL/s, an International Prostate Symptom Score (IPSS) of 7 or higher, or urinary retention. 

The preoperative patient evaluation consisted of a comprehensive assessment that involved a physical examination, including a digital rectal examination, measurement of prostate-specific antigen (PSA) levels, estimation of PV using abdominal ultrasound, uroflowmetry to quantify maximum urine flow rate (Qmax), measurement of postvoid residual urine (PVR), urinalysis, and urine culture. Furthermore, before surgical indication, it was always necessary to provide symptom questionnaires such as IPSS and Quality of Life (QoL) as in follow-up visits.

### 2.2. Inclusion Criteria

We conducted a retrospective analysis to identify two distinct groups of patients. The first group consisted entirely of patients who received TLP, while the second group included all patients who underwent TURP during the study period. For both groups, we collected and recorded preoperative, intraoperative, and postoperative data. The matching process primarily examined PV and ASA scores as the key preoperative indicators to find patients with similar characteristics.

### 2.3. Exclusion Criteria

To limit a possible result bias, we decided to exclude individuals who had incidentally discovered prostate cancer and subsequently received treatment. In addition, characteristics such as the urodynamic evidence of a neurogenic bladder or recorded detrusor hypoactivity or hyperactivity, as well as a past history of prostate or urethral surgery or prostate cancer, were also taken into account as factors to be excluded.

### 2.4. Surgical Technique

All surgical interventions were completed by a single experienced surgeon using the same technique and devices.

The bipolar resections (Olympus Plasma + system, Olympus America, Melville, NY, USA) of the prostate were made according to standard procedures. The power level for cutting was 100 W, whereas the power setting for coagulation was 120 W.

The combined technique (TLP) was performed using a Thulium: YAG laser Cyber TM (Quanta System, Samarate, Italy) with a wavelength of 2010 nm and an 800 μm optical, bare-ended reusable laser fiber introduced via a 26 F continuous flow Karl Storz resectoscope. The power settings of the laser device for the enucleation and coagulation of the prostatic tissue were 190 W and 50 W, respectively.

The process commenced with a cystoscopy to assess the bladder and ureteral orifices, followed by an examination of the prostatic lobes. A horizontal cut was made above the verumontanum to indicate the lower boundary. Two linear cuts were created at the positions of 5 and 7 o’clock, reaching up to the anterior transverse cut. The cutting fiber was shifted in a semi-circular manner from the verumontanum to the bladder neck. The median lobe was initially ablated, beginning at the verumontanum and extending to the bladder neck. Next, the lateral lobes were subjected to vaporization, and then a bipolar resection was carried out to reach the prostate capsule. Subsequently, the prostate tissue was extracted via the resectoscope. Ultimately, bipolar plasmavaporization was employed to flatten the prostatic fossa, so achieving regularity.

For both procedures, isotonic saline, at room temperature, was used as an irrigation fluid throughout all interventions. Following surgery, all patients had a 22 F three-way Dufour tip Foley catheter with continuous bladder irrigation.

### 2.5. Immediate and Long-Term Comparison

We created a medical database to document intraoperative and follow-up variables at 3, 6, 12, and 24 months after surgery. The use of this clinical database facilitated the assessment of immediate surgical outcomes such as operative time, complication rate, and length of hospital stay. The amount of blood lost during surgery was determined by calculating the decrease in hemoglobin (Hb) levels. This was performed by measuring the difference between preoperative and postoperative Hb levels within a time frame of 12–24 h. An analysis was performed to compare the results of early and late follow-up using objective measures (such as PSA decrease, Qmax, and PVR) and validated symptom questionnaires, including the IPSS and the QoL index. The incidence of surgical complications was recorded.

### 2.6. Statistical Analysis

Counts and percentages were used to present qualitative results. Means and standard deviations or medians and quartiles (1 and 3) were used to present quantitative results. We matched TLP patients with bipolar TURP patients to have the same ASA group (1, 2 versus 3), and a similar baseline prostatic volume (in the range of 10 mL), using the MatchIt R package version 4.5.5. [14], resulting in two paired groups of 60 patients each. Comparisons between the paired groups regarding binary data were performed with Mc Nemar test, while those for nominal data were performed with the Stewart–Maxwell test. Comparisons between the paired groups regarding normally distributed data were performed with the *t*-test for paired samples, while those for data not following the normal distribution were performed with the Wilcoxon signed-rank test. For all analyses, the two-tailed *p*-value and the 0.05 levels of significance were used. All statistical analyses were carried out with the R environment for statistical computing and graphics (R Foundation for Statistical Computing, Vienna, Austria), version 4.3.2. [15].

## 3. Results

From March 2018 to December 2021, data from 60 patients who underwent TLP were compared with 60 similar patients who underwent bipolar TURP during the same period. Baseline patient characteristics are shown in Table 1. Age, PV (including its subcategories), ASA score, and preoperative functional variables (such as QoL index, Qmax, and PVR) were clinically very similar. There were significant differences concerning PV and IPSS scores, but as observed, the median and interquartile ranges are almost identical. These differences are due to the matching that allowed 10 mL difference regarding PV between groups. The number of patients with indwelling catheters was higher in the TLP group, but did not reach statistical significance.

Intraoperative characteristics and outcomes of the two match-paired patient groups are shown in Table 2. There was a shorter operative time in favor of TLP, but not reaching statistical significance. Median hemoglobin drop and median sodium drop after surgery were statistically significantly lower in TLP compared to the bipolar TURP. Catheterization and hospital stay were similar.

There were more early and late complications in the bipolar TURP group (13, 21.67%) compared to the TLP group (12, 20%), but the differences did not reach statistical significance level (Table 3). There were no capsular perforations, reoperations for bleeding, and persistent stress incontinence at 6 months in both groups. There were no late complications in the TLP group, while there were three urethral strictures and one bladder neck contracture in the bipolar TURP group.

### Follow-Up Data

**Figure 1:**
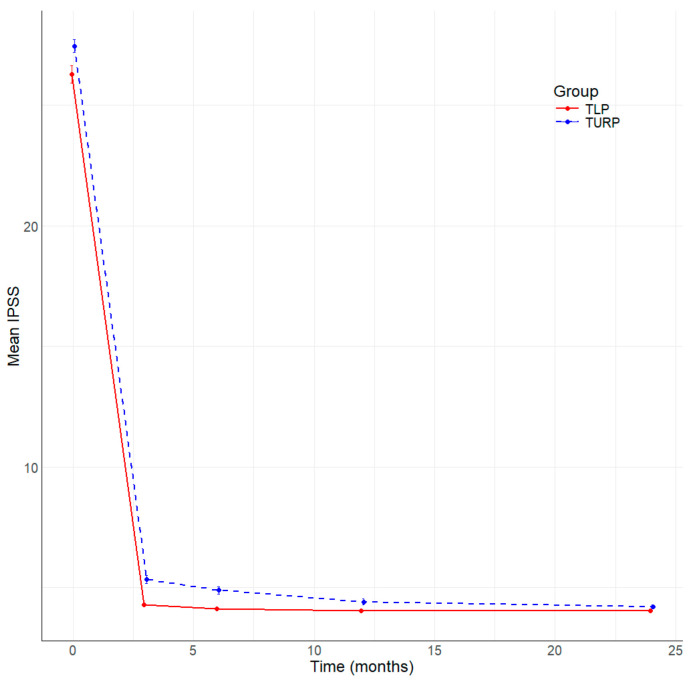
Evolution of IPSS by time (months) and group.

**Figure 2:**
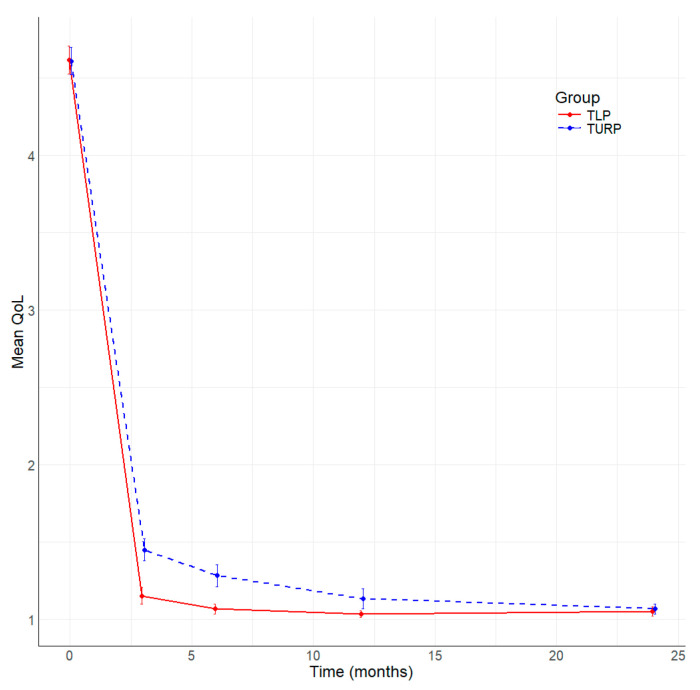
Evolution of QoL by time (months) and group.

**Figure 3:**
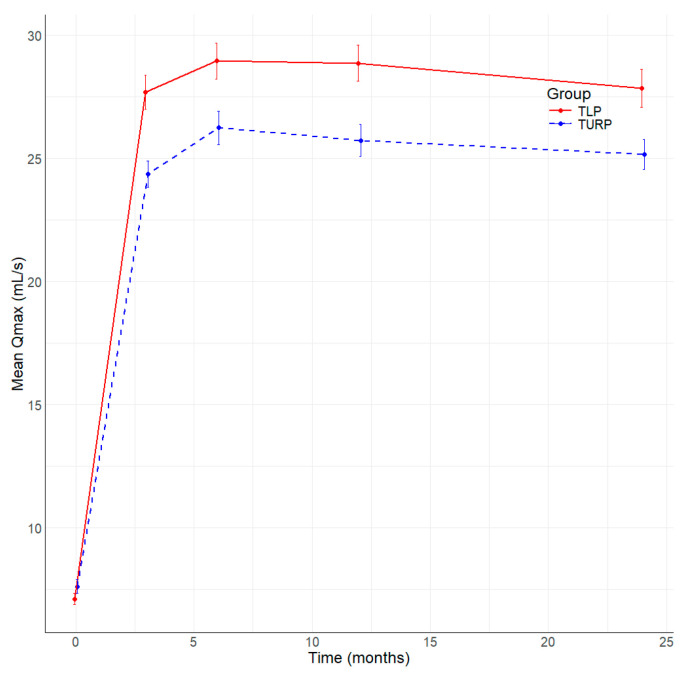
Evolution of Qmax (mL/s) by time (months) and group.

**Figure 4:**
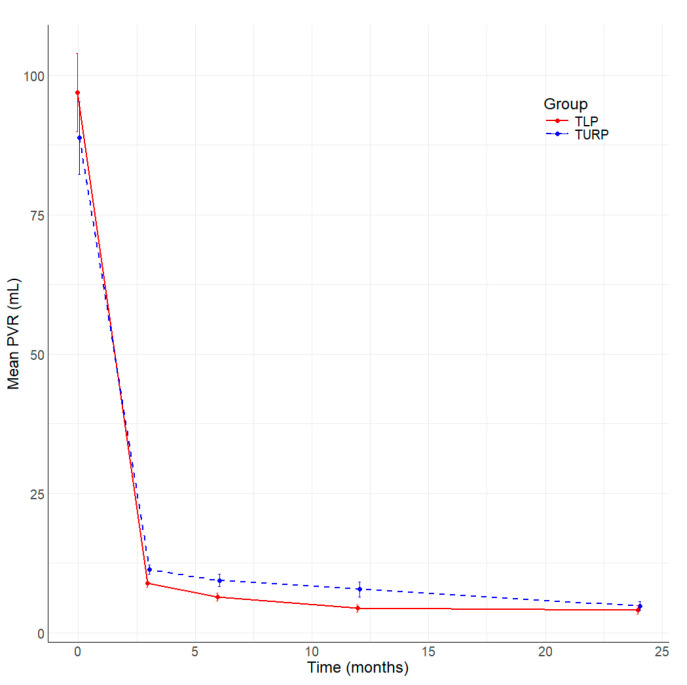
Evolution of PVR (mL) by time (months) and group.

All patients included in the study underwent complete follow-up at 3, 6, 12, and 24 months after surgery. Table 4 summarizes the main parameters evaluated in this study. The resolution of symptoms is well represented by the significant improvement in the median IPSS score at 3, 6, and 12 months (Figure 1), and better IPSS at 24 months without being statistically significant in TLP compared to bipolar TURP. The QoL is better in the TLP group than in the bipolar TURP (Figure 2) between 3 and 24 months, the difference being statistically significant at 3 and 6 months. The Qmax had statistically significantly better values TLP group compared to bipolar TURP (Figure 3) for all measurements between 3 and 24 months. The PVR is smaller in the TLP group compared to bipolar TURP (Figure 4) between 3 and 24 months, the difference being statistically significant at 3, 6, and 12 months.

**Figure 5:**
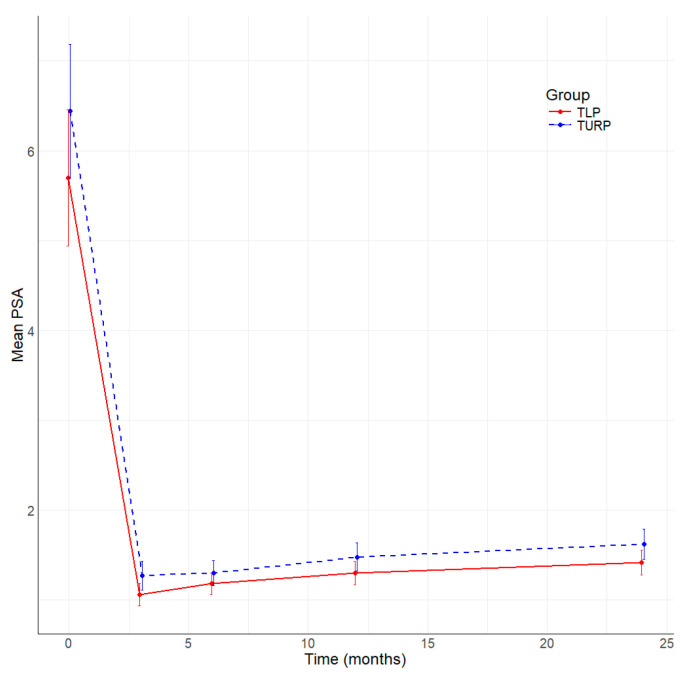
Evolution of PSA by time (months) and group.

The median PSA drop after surgery was 73.9% in the TLP group versus 76.17% in the bipolar TURP group, and the difference was not statistically significant (Figure 5).

## 4. Discussion

The gold standard therapy for medium-sized prostates is still TURP [16]. Monopolar TURP is linked to a significant incidence of perioperative complications, with a rate of 11.1%. The most notable complications include the inability to urinate (5.8%), the requirement for further surgical intervention (5.6%), severe urinary tract infection (3.6%), bleeding that necessitates blood transfusions (2.9%), and transurethral resection syndrome (1.4%) [17]. On the other hand, utilizing bipolar TURP with normal saline decreases the likelihood of the TUR syndrome and enables longer surgical durations, hence facilitating the potential for operating on greater prostate sizes [2]. Open prostatectomy (OP) has traditionally been used for high-volume prostates, although it is currently being substituted with a robotic approach [18] or laser technology. HoLEP, introduced in 1998, has been a major breakthrough in the field of surgery because it is not limited by size [19,20]. In addition, it has demonstrated favorable functional outcomes in long-term follow-up assessments [21]. Thulium laser was subsequently introduced in 2005 [22]. 

In recent decades, several surgical techniques have been developed using the Thulium laser’s continuous wave mode. ThuVARP, also known as ‘VapoResection’, involves removing prostatic tissue in a similar manner to the TURP resection, but with the addition of concomitant vaporization. This combination results in higher tissue ablation rates compared to TURP and eliminates the need for morcellation [23]. Xia et al. proposed a technique called the Thulium laser resection of the prostate–tangerine technique (ThuLRP). The technique involves creating prostatic chips in a tangerine-like fashion through a combination of semicircular and transverse incisions. These prostatic chips can also be removed using the resectoscope sheath [22]. The thulium laser vaporization of the prostate (ThuVAP) was first described by Mattioli et al. The procedure involves using a painting technique to ablate the adenoma. The use of high-output power generally results in faster removal of the adenoma [24]. The technique of “vapo-enucleation” was first described by Bach et al. in 2009, and involves enucleating the prostatic lobes along the prostatic capsule and concomitantly vaporizing the prostatic tissue using a medium-high power laser. After the enucleation, the tissue was morcellated inside the bladder [25]. In 2010, Herrmann et al. introduced the Thulium laser enucleation procedure (ThuLEP). This technique involves using the thulium laser to incise the mucosa of the apex to the prostatic capsule followed by blunt dissection of the adenoma from the prostatic capsule using the sheath of the endoscope, similarly to the surgeon’s finger in OP. Low-power laser is used for the precise coagulation of the crossing vessels during the blunt dissection. A morcellator is needed to retrieve the enucleated adenoma [26]. For both enucleation techniques, the objective is to achieve anatomical enucleation.

ThuVARP is a well-researched alternative to TURP. A randomized controlled trial (RCT) comparing ThuVARP with monopolar TURP revealed no statistically significant difference in the effectiveness or the rate of reoperation (2.1% versus 4.1%) under long-term monitoring [27]. A meta-analysis found that performing ThuVARP at 70 W resulted in lengthier surgical procedures, shorter periods of catheterization and hospitalization, and reduced blood loss. However, there was no significant difference in the transfusion rates or other short-term complication rates compared to TURP [28]. An extensive trial on ThuVARP, conducted across many centers and comprising 2216 patients, demonstrated long-lasting improvements in IPSS, QoL, Qmax, and PVR volume during the 8-year follow-up period [29]. In a multicenter, randomized, controlled, parallel-group trial, patients undergoing TURP or ThuVARP had similar surgical outcomes in terms of complications, blood transfusion rates, and hospital stay. However, TURP was found to be superior to ThuVARP in terms of Qmax [30].

As we continue to evaluate the efficacy and efficiency of the TLP technique, it is important to consider broader trends in the surgical treatment of BPH. A recent study [31] has shown a significant increase in the adoption of novel surgical treatments over the past decade, with these treatments generally having a higher cost but potentially reducing the likelihood of surgical retreatment compared to traditional procedures. These findings underscore the importance of continuous innovation in surgical techniques, such as our TLP approach, which seeks to optimize both patient outcomes and cost-effectiveness. In the area of research aiming to improve surgical procedures for BPH, a recent study [32] examined the differences between the en-bloc, three-lobe, and two-lobe techniques of thulium laser enucleation and found that the en-bloc and two-lobe techniques may offer efficiency benefits concerning operative time and energy delivered, although they did not show significant improvements in short-term functional outcomes. This comparison may be useful to contextualize the benefits of our TLP technique versus other surgical options and to highlight how technique selection may be influenced by surgeon preference and skill, as well as specific patient needs.

Two studies were conducted that compared the efficacy of the combined techniques with that of bipolar TURP in the treatment of large prostates. However, the follow-up periods were relatively short. Xie et al. evaluated the combination of using a Thulium laser and transurethral bipolar plasmakinetic resection of the prostate. The combined technique was found to be superior in terms of surgical duration, intraoperative blood loss, hospital stay, and postoperative complications. There were no statistically significant differences in IPPS score, QoL, Qmax, and PVR [13]. Huang et al. conducted an evaluation of the combination of Thulium laser incision and bipolar TURP. The study reported significant differences in the resection efficiency in favor of the combined technique.

This study aimed to combine the advantages of Thulium laser, bipolar TURP, and PKVP while eliminating their respective drawbacks. To this end, we introduced a multimodal treatment for BPH endoscopic surgery.

Our data demonstrated that this technique resulted in a lower operative time, but was not statistically significant while yielding equivalent postoperative outcomes regarding IPSS, QoL, and PVR at 24-month follow-up. The non-significant result regarding the operative time might be explained by the relatively smaller sample size. In a study by Huang et al., the operative time was shorter in the combined technique group, but not statistically significant (67.3 ± 24.4 versus 73.3 ± 25.1, *p* = 0.094), but longer than our operative time [12]. In contrast, the findings of Xie et al. indicated a statistically significant reduction in surgical duration [13]. The operative time included the time wasted in changing instruments. This time was not recorded separately. Nevertheless, it was very short, less than a minute, since all the equipment was already prepared at the beginning of the surgery or during the thulium vaporization. However, Qmax was significantly statistically better at all follow-up visits compared to TURP. The initial step of the Thulium vaporization of the prostatic tissue allows for the safe and efficient performance of the procedure in a clean environment. Intraoperative bleeding may impede the efficacy of endoscopic resection due to suboptimal visualization and may necessitate a prolonged operative time due to the necessity to cease the procedure and perform hemostasis. The Thulium laser is an effective tool for achieving hemostasis during tissue vaporization procedures.

Our comparative analysis of the two surgical techniques revealed a lower hemoglobin decline in favor of the multimodal treatment of 0.3 statistically significant (*p* < 0.001). Furthermore, none of our patients irrespective of the surgical technique required a blood transfusion after surgery. Xia et al. reported a statistically significant reduction in blood loss, whereas Huang et al. observed no significant differences in the decline of modified hemoglobin [12,13].

There was no statistical difference regarding catheterization and hospital stay time.

The complication rate was comparable, and the incidence of complications was very low with both techniques (overall 20% in the TLP group versus 21.67% in the bipolar TURP group). We report no late complications in the TLP group. The lower complication rate is due to the benefits of using thulium laser technology. Other combined techniques also reported no significant differences in the overall complication rate, length of hospital stay, and catheterization [12].

This work has some limitations: its retrospective and unblinded nature. As a single-center study, the outcomes may not be generalizable to broader clinical settings or different patient populations. The study is non-randomized and observational, which may introduce biases (selection of confounding) that could affect the results. Causal affirmations cannot be drawn from this type of design. Concerning blood loss, there is always the possibility of underestimating or overestimating the amount of fluids given during the operation. Also, it was not possible to make a comparison between erectile and sexual function after surgery.

However, we think that a matched-pair comparative research design is a useful way to choose patients with similar preoperative features, even in the absence of the power of a randomized prospective trial.

## 5. Conclusions

The results show that both procedures are equally effective and safe in treating symptomatic BPH in terms of IPSS, QoL, and PVR at 24-month follow-up. TLP offered some advantages over bipolar TURP in terms of hemoglobin and sodium reduction, and higher Qmax at 24 months. Future research should concentrate on the specifics of larger cohorts, urodynamic improvement, and long-term outcomes.

## Figures and Tables

**Table 1:** Preoperative characteristics of the patients considered in the study.

	TLP (*n* = 60)	Bipolar TURP (*n* = 60)	*p* Value
Age, (years), mean (SD)	64.38 (7.03)	65.17 (7.64)	0.508
Age in classes, *n* (%)			0.603
<60 years	14 (23.33)	15 (25)	
60–70 years	34 (56.67)	29 (48.33)	
>70 years	12 (20)	16 (26.67)	
ASA score in classes, *n* (%)	43 (71.67)	43 (71.67)	1
1 + 2	17 (28.33)	17 (28.33)	
3			
PV, mL, median (IQR)	70 (60–75)	70 (60–75)	0.01
PV in classes			1
<60	9 (15)	9 (15)	
60–80	39 (65)	39 (65)	
80–100	1 (1.67)	1 (1.67)	
100–120	3 (5)	3 (5)	
>120	8 (13.33)	8 (13.33)	
Indwelling catheterization, *n* (%)	12 (20)	9 (15)	0.635
IPSS, median (IQR)	27 (25–28)	27 (26–28)	0.038
QoL, median (IQR)	4 (4–5)	5 (4–5)	0.882
PSA (ng/mL), median (IQR)	4.43 (2.58–6.12)	4.7 (3.55–7.28)	0.531
Qmax (mL/s), median (IQR)	7 (5.85–8.25)	7.4 (6.6–9.35)	0.218
PVR (mL), median (IQR)	80 (68.5–105)	80 (68–90)	0.237

TLP, multimodal treatment; TURP, transurethral resection of the prostate; IQR, interquartile range. ASA score, American Society of Anesthesiologists classification; PV, prostate volume, IPSS, International Prostate Symptom Score; PSA, prostate-specific antigen; PVR, postvoid residual urine; Qmax, maximum urinary flow rate; QoL, quality of life.

**Table 2:** Intraoperative characteristics and outcomes of the two match-paired patient groups.

	TLP (*n* = 60)	Bipolar TURP (*n* = 60)	*p* Value
Operative time, min, median (IQR)	42 (34.75–50)	45 (35–50)	0.402
Hemoglobin drop, g/dL, median (IQR)	−0.3 (−0.4–−0.2)	−0.6 (−0.75–−0.4)	<0.001
Sodium drop, mmol/L, median (IQR)	−0.3 (−0.7–0)	−0.7 (−1.2–−0.3)	<0.001
Catheterization time, days, median (IQR)	7 (6.25–7)	7 (7–7)	0.053
Hospital stay, days, median (IQR)	2 (1–2)	2 (1.5–2)	0.917

TLP, multimodal treatment; TURP, transurethral resection of the prostate; IQR, interquartile range.

**Table 3:** Early and late surgical complications occur in our TLP and bipolar TURP prostate series.

Complications	TLP (*n* = 60)	Bipolar TURP (*n* = 60)	*p* Value
Early complications	12 (20)	11 (18.33)	1
Capsular perforation	0 (0)	0 (0)	1
Reoperation for bleeding	0 (0)	0 (0)	1
Early acute urinary retention	1 (1.67)	3 (5)	0.5
Cloth retention	1 (1.67)	1 (1.67)	1
Urinary irritation/UTI	9 (15)	8 (13.33)	1
Transient urinary incontinence (at 3 mo)	3 (5)	5 (8.33)	0.727
Late complications	0	4 (6.67)	1
Urethral stricture	0 (0)	3 (5)	0.248
Bladder neck contracture	0 (0)	1 (1.67)	1
Persistent stress incontinence (at 6 mo)	0 (0)	0 (0)	1
BPH recurrence	0 (0)	1 (1.67)	1
Early and late complications (%)	12 (20)	13 (21.67)	1

The table presents the number of patients (%) that had specific types of complications; A patient can have multiple early or late complications, or both early and late complications; BPH, benign hyperplasia of the prostate, TLP, multimodal treatment; TURP, transurethral resection of the prostate.

**Table 4:** Follow-up the early and late functional outcomes in the two groups at 3, 6, 12, and 24 months after surgery.

	TLP (*n* = 60)	Bipolar TURP (*n* = 60)	*p* Value
3-month follow-up			
IPSS, median (IQR)	4 (4–4)	5 (4–6)	<0.001
QoL (range)	1 (1–1)	1 (1–2)	0.001
Qmax, mL/s, median (IQR)	27.8 (26.18–29.88)	25.95 (20.3–27.8)	<0.001
PVR, mL, median (IQR)	8.3 (4.85–12)	10.6 (7–14)	0.042
6-month follow-up			
IPSS, median (IQR)	4 (4–4)	4 (4–5)	<0.001
QoL (range)	1 (1–1)	1 (1–1)	0.01
Qmax, mL/s, median (IQR)	29.25 (26.42–32.6)	27.1 (23.58–29.42)	0.006
PVR, mL, median (IQR)	6 (2.5–10)	8 (2.45–14)	0.034
12-month follow-up			
IPSS, median (IQR)	4 (4–4)	4 (4–4)	0.004
QoL (range)	1 (1–1)	1 (1–1)	0.188
Qmax, mL/s, median (IQR)	28.75 (25.85–32.73)	26.2 (22.87–28.9)	0.001
PVR, mL, median (IQR)	4 (0–7)	6 (2–9)	0.016
24-month follow-up			
IPSS, median (IQR)	4 (4–4)	4 (4–4)	0.083
QoL (range)	1 (1–1)	1 (1–1)	0.777
Qmax, mL/s, median (IQR)	28.6 (24.6–31.72)	26 (22.47–28.42)	0.005
PVR, mL, median (IQR)	2.6 (0–5.95)	3.1 (0–7.15)	0.279
PSA drop (%), median (IQR)	73.92 (55.79–81.36)	76.17 (60.85–83.83)	0.578

TLP, multimodal treatment; TURP, transurethral resection of the prostate; IQR, interquartile range; IPSS, International Prostate Symptom Score; PSA, prostate-specific antigen; PVR, postvoid residual urine; Qmax, maximum urinary flow rate; QoL, quality of life.

## Data Availability

The data that support the conclusion of this study will be made available by the authors upon reasonable request.
